# Long noncoding RNA CAR10 promotes lung adenocarcinoma metastasis via miR-203/30/SNAI axis

**DOI:** 10.1038/s41388-018-0645-x

**Published:** 2019-01-07

**Authors:** Xiaolu Ge, Gui-yuan Li, Lin Jiang, Liqing Jia, Zhezhe Zhang, Xiaoling Li, Ranran Wang, Ming Zhou, Yanhong Zhou, Zhaoyang Zeng, Juanjuan Xiang, Zheng Li

**Affiliations:** 1 The Key Laboratory of Carcinogenesis of the Chinese Ministry of Health, Hunan Cancer Hospital and the Affiliated Cancer Hospital of Xiangya School of Medicine, Central South University, Changsha, Hunan China; 2 Cancer Research Institute, School of Basic Medical Sciences, Central South University, Changsha, Hunan China; 3 The Key Laboratory of Carcinogenesis and Cancer Invasion of the Chinese Ministry of Education, Cancer Research Institute, Central South University, Changsha, Hunan China; 4 Department of Thoracic and Cardiovascular Surgery, The Second Xiangya Hospital, Central South University, Changsha, Hunan 410011 China; 5 High Resolution Mass Spectrometry Laboratory of Advanced Research Centre, Central South University, Changsha, China

**Keywords:** Non-small-cell lung cancer, Metastasis, Non-coding RNAs

## Abstract

Long noncoding RNAs (lncRNAs) play an important role in lung adenocarcinoma (LUAD) metastasis. Here, we found that lncRNA chromatin-associated RNA 10 (CAR10) was upregulated in the tumor tissue of patients with LUAD and enhanced tumor metastasis in vitro and in vivo. Mechanistically, CAR10 induced epithelial-to-mesenchymal transition (EMT) by directly binding with miR-30 and miR-203 and then regulating the expression of *SNAI1* and *SNAI2*. CAR10 overexpression was positively correlated with a poor prognosis in LUAD patients, whereas overexpression of both CAR10 and *SNAI* was correlated with even worse clinical outcomes. In conclusion, the CAR10/miR-30/203/*SNAI* axis is a novel and potential therapeutic target for LUAD.

## Introduction

Lung cancer is the most common cause of cancer-related deaths in China and worldwide [1, 2]. Lung adenocarcinoma (LUAD) is the most prominent histological subtype of non-small-cell lung cancer (NSCLC) with a high rate of mortality and metastasis [3]. Despite the advances in the research on epidermal growth factor receptor (*EGFR*) gene mutations, echinoderm microtubule-associated protein-like 4 and anaplastic lymphoma kinase (*EML4-ALK*) gene rearrangement, and the associated targeted therapy with tyrosine kinase inhibitors and crizotinib, respectively, the prognosis of patients with late-stage LUAD remains unfavorable with a 5-year survival rate of 4% [2, 4, 5]. Studies on driving gene alterations involved in LUAD progression represent an area of continuing research intended to find a diagnostic biomarker and therapeutic targets of LUAD.

Long noncoding RNAs (lncRNAs) are a class of transcripts longer than 200 nucleotides with limited protein-coding potential. Recently, some reports showed that lncRNAs are abnormally expressed in LUAD patients and regulate tumor metastasis processes [6, 7]. For example, metastasis-associated lung adenocarcinoma transcript 1 (MALAT1) has been identified as a marker for monitoring metastasis progression and predicting survival in non-small-cell lung cancer [8]. MALAT1 also increased brain and bone metastasis of LUAD cells [9, 10]. Additionally, lncRNA ANRIL encoded in chromosomal region 9p21 and in the opposite direction of the INK4B-ARF-INK4A gene cluster is upregulated in NSCLC tissues and is positively associated with metastasis and poor prognosis [11, 12]. ANRIL plays an oncogenic role in LUAD by inhibiting *P21* and *KLF2* transcription [13].

In this study, we profiled matched tissue samples from LUAD patients with HTA 2.0 microarray analysis and found that lncRNA chromatin-associated RNA 10 (CAR10) is clearly overexpressed in LUAD tissues. CAR10 was first identified during sequencing of chromatin-associated RNAs (CARs) from human fibroblasts that regulate the expression of neighboring genes in *cis* [14]. CAR10 is upregulated in leiomyomas and may contribute to the pathogenesis of uterine leiomyomas [15]. CAR10 is upregulated by carcinogen dibenz[a,h]anthracene that increases proliferation of lung cancer cells by binding transcription factor Y-box-binding protein 1 (YB-1) [16]. However, whether CAR10 can promote the metastasis of LUAD and the underlying molecular mechanisms remains unclear.

In this report, we demonstrated that CAR10 accelerates tumor growth and promotes metastasis of LUAD. CAR10 was shown to promote epithelial-to-mesenchymal transition (EMT) of LUAD cells through sponging miR-30 and miR-203, leading to upregulation of *SNAI1* and *SNAI2*. Finally, elevated CAR10 expression was found to correlate with a poor prognosis, whereas simultaneous overexpression of CAR10 and *SNAI1* and/or *SNAI2* correlated with shorter survival time among LUAD patients. Receiver- operating characteristic curve (ROC) analysis revealed important diagnostic values of CAR10 for LUAD. CAR10 is a good candidate for a molecular marker for LUAD diagnosis and treatment.

## Results

### CAR10 was upregulated in LUAD

**Fig. 1:**
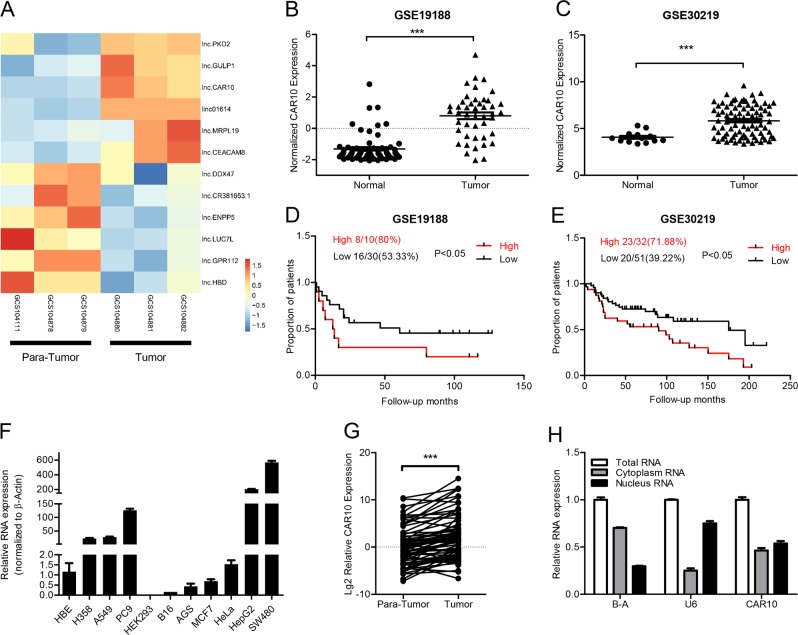
CAR10 upregulation in lung adenocarcinoma cell lines and tissues. **a** Hierarchical clustering analysis of the top 12 differentially expressed lncRNAs between lung adenocarcinoma samples (tumor) and paired adjacent lung tissue (para-tumor) samples. Red color represents upregulated lncRNAs, and blue color represents downregulated genes in lung adenocarcinoma samples. **b**, **c** CAR10 expression in human LUAD tissue samples and paired adjacent lung tissue samples in the analysis of GSE19188 and GSE30219. **d**, **e** Kaplan–Meier survival analysis of the correlation between CAR10 expression and overall survival of patients with LUAD (*P* < 0.05). **f** The expression of CAR10 in LUAD cells and other cancer cells compared to human bronchial epithelial (HBE) cells. Data are presented as the mean ± SEM of three independent experiments. **g** CAR10 expression was analyzed by qRT-PCR in matched tumor samples (*n* = 74) compared with para-tumor samples (*n* = 74). Two-tailed paired Student’s t test. **h** Relative CAR10 expression levels in the cytoplasm and nucleus of A549 cells. Data are presented as the mean ± SEM of three independent experiments, two-tailed Student’s *t* test. *P < 0.05, **P < 0.01, and ***P < 0.001. NS no statistical significance

To investigate the lncRNA expression profile in LUAD tissues, three pairs of LUAD tissue samples (three LUAD tissue samples and three matched adjacent lung tissue samples) were analyzed with human transcriptome microarray Affymetrix^®^ HTA 2.0. Via criteria log2FC (fold change) > 1.5 or < −1.5 and *P* value < 0.05, 473 ncRNAs were identified whose expression differed between LUAD and the adjacent lung tissue (Supplementary Figure 1A and 1B). The protein-coding potential of the top 16 differentially expressed ncRNAs was tested with five protein-coding potential metrics (Supplementary Table 2 and 3). A heatmap of 12 of the most upregulated and downregulated ncRNAs was shown in Fig. 1a. Some lncRNAs overlapped with CDS regions of other genes, but we were interested only in upregulated intergenic lncRNAs: CAR10 and lnc01614. By means of the NOCODE database, we found the expression of lnc01614 to be almost zero in lung tissues, and CAR10 had a low expression level (Supplementary Figure 1C). Analysis of datasets GSE19188 and GSE30219 [17, 18] revealed found CAR10 expression to be significantly increased in LUAD tissues as compared with the matched para-tumor tissues (Fig. 1b, c). Moreover, increased CAR10 expression in LUAD tissues clearly correlated with a poor prognosis in LUAD patients (Fig. 1d, e). CAR10 turned out to be upregulated in LUAD cell lines (A549, PC9, and H358) and other tumor cell lines (HepG2 and SW480) compared with human bronchial epithelial cells (HBE) (Fig. 1f). CAR10 was found to be located in both the cytoplasmic and nuclear compartments as evidenced by cell fractionation (Fig. 1h). Next, we examined the expression of CAR10 in 74 paired LUAD samples, including four paired samples with metastasis tissues. CAR10 was found to be overexpressed in LUAD (Fig. 1g) with higher expression in metastatic tissues (Supplementary Figure 1D). Thus, we focused on the role and mechanisms of action of CAR10 in LUAD metastasis.

### CAR10 promoted a metastatic phenotype in LUAD cells

**Fig. 2:**
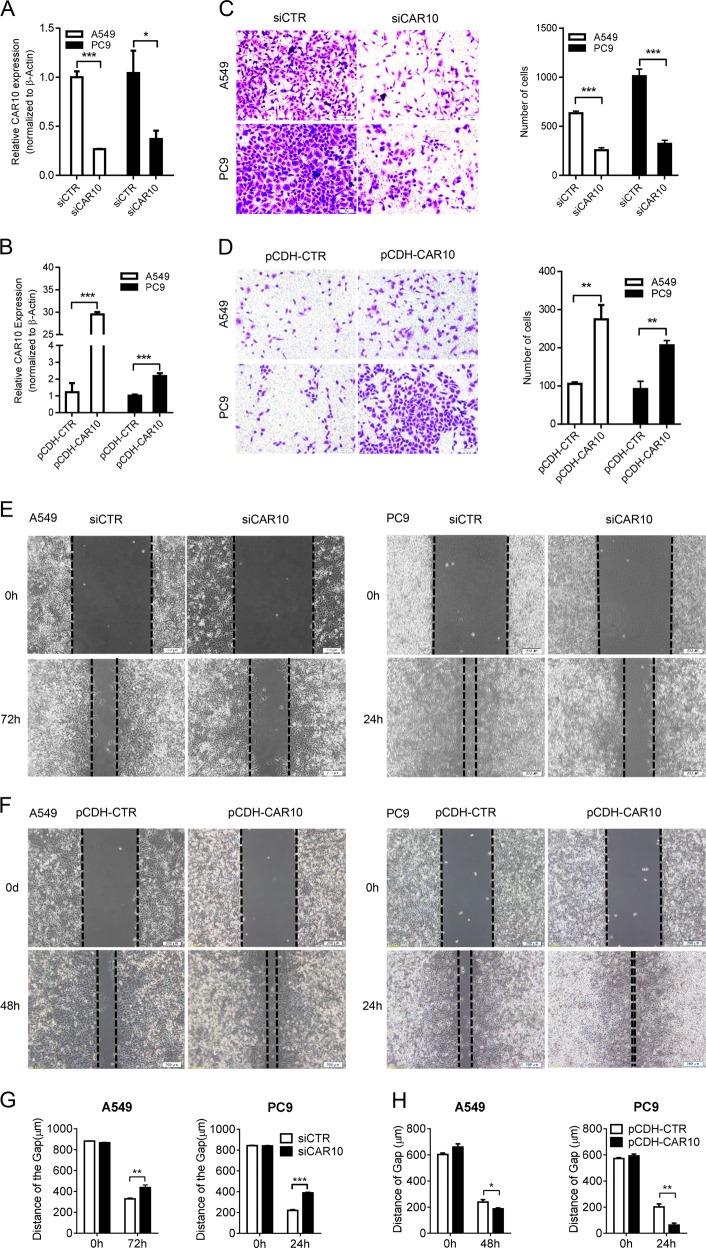
Effects of CAR10 on invasion and migration of LUAD cells in vitro. **a** Efficiency of the CAR10 knockdown in A549 and PC9 cells using the independent lncRNA Smart Silencer of CAR10. Data are presented as the mean ± SEM of three independent experiments, two-tailed Student’s *t* test. **b** Overexpression of CAR10 (pCDH-CAR10) in A549 and PC9 cells using a lentiviral vector to stably increase CAR10 expression in infected LUAD cells. Mean ± SEM; *n* = 3 independent experiments, two-tailed Student’s *t* test. **c**, **d** The invasive potential of silenced CAR10 or pCDH-CAR10 A549 and PC9 cells together with their controls was analyzed by a transwell assay. Scale bar: 100 μm. Number of invasive cells was shown as mean ± SEM; *n* = 3 independent experiments, two-tailed Student’s *t* test. **e**–**h** The migratory potential of CAR10 knockdown or pCDH-CAR10 A549 and PC9 cells together with their controls was analyzed in a wound-healing assay. Scale bar: 200 μM. Distance of the gap was shown as mean ± SEM; *n* = 3 independent experiments, two-tailed Student’s *t* test. *P < 0.05, **P < 0.01, and ***P < 0.001. NS no statistical significance

In order to investigate the role of CAR10 in LUAD cells, the expression of CAR10 was knocked down using lncRNA Smart Silencer (siCAR10) in cell lines A549 and PC9. The lncRNA Smart Silencer is a mixture of three siRNAs and three antisense oligonucleotides (ASOs), which more efficiently knock down CAR10 expression (below 50%) in both cytoplasmic and nuclear fractions as compared with the use of a single siRNA (Fig. 2a, Supplementary Figure 2A). Next, we tested whether CAR10 could affect the migration and invasiveness of LUAD cells. Transwell and wound-healing assays indicated that compared with the cells treated with control Smart Silencer, suppression of CAR10 expression attenuated the invasive and migratory abilities of A549 and PC9 cells (Fig. 2b–d). Conversely exogenous expression of CAR10 promoted the migration and invasiveness of LUAD cells compared with the cells transfected with the control vector (Fig. 2e–h).

The CAR10 knockdown inhibited cell growth and colony formation of A549 and PC9 cell lines. Increasing the expression of CAR10 had an opposite effect of promoting the proliferation of cells (Supplementary Figure 2). These results indicated that CAR10 plays an important role in LUAD progression, specifically in invasion and migration.

### CAR10 knockout inhibited LUAD cell proliferation and metastasis in nude mice

**Fig. 3:**
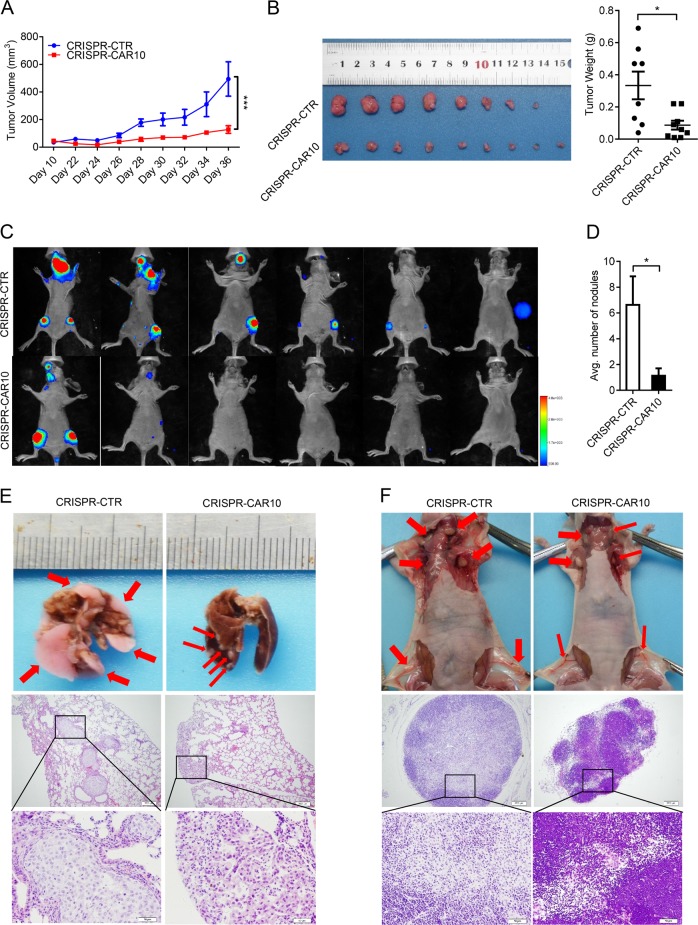
CAR10 silencing inhibited tumor initiation/growth and metastasis in vivo. **a**, **b** The knockout of CAR10 via a CRISPR/Cas9 system inhibited the growth of A549 cells and subcutaneously transplanted the xenograft into BALB/c nude mice. Data are shown as mean ± SEM; *n* = 8–9 mice per group, two-tailed Student’s *t* test. **a** Tumor formation was monitored at the indicated time points. **b** Overall tumor growth of the xenografts, and tumor weights were recorded. **c** CRISPR-CTR or CRISPR-CAR10 A549 cells were intravenously injected into nude mice. Luciferase signal intensities of each group were examined at 8 weeks. **d** The number of metastatic nodules in the lungs is shown as mean ± SEM (*n* = 6), using two-tailed Student’s *t* test. **e** Representative lungs and hematoxylin and eosin-stained images presented to show metastases in the lungs (scale bar, 200 μm and 50 μm). **f** Representative lymph nodes and hematoxylin and eosin-stained images of lymph nodes isolated from metastatic tumor mouse models. *P<0.05, **P<0.01, and ***P <0.001. NS no statistical significance

We designed a pair of small-guide RNAs (sgRNAs) to knock out CAR10 in A549 cells by means of the CRISPR/Cas9 system to determine whether CAR10 can promote LUAD progression in vivo (Supplementary Figure 3A and 3B). We chose one colony, in which expression of CAR10 was downregulated to almost 80% (Supplementary Figure 3C). To monitor tumor metastasis in vivo, A549 CRISPR-CTR and CRISPR-CAR10 cells with stable luciferase expression were established. The transwell assay showed that CRISPR-CAR10 effectively inhibited the invasive ability of A549 cells (Supplementary Figure 3D). Using a xenograft model, we found that knocking out CAR10 obviously inhibited the tumor growth and yielded significantly smaller tumors after 5 weeks, as compared with A549 CRISPR-CTR cells (Fig. 3a, b and Supplementary Figure 3E). Then, we evaluated invasiveness and metastasis inhibition by the knockout of CAR10 in a tail vein injection model. Nude mice were injected via the tail vein with 4 × 10^6^ cells, and then bioluminescence was examined via an imaging system to monitor the locations and growth of tumor metastasis foci. Seven weeks after injection, we found that A549 CRISPR-CAR10 cells produced significantly weaker lymphatic metastasis as compared with A549 CRISPR-CTR cells (Fig. 3c). Next, histological examination of lung and lymph nodes revealed fewer lung nodules and lymphatic metastases in mice injected with A549 CRISPR-CAR10 cells than in mice injected with A549 CRISPR-CTR cells (Fig. 3d–f). These results indicated that the CAR10 knockout inhibited LUAD proliferation and metastasis in vivo.

### CAR10 induced EMT by upregulating *SNAI1* and *SNAI2*

**Fig. 4:**
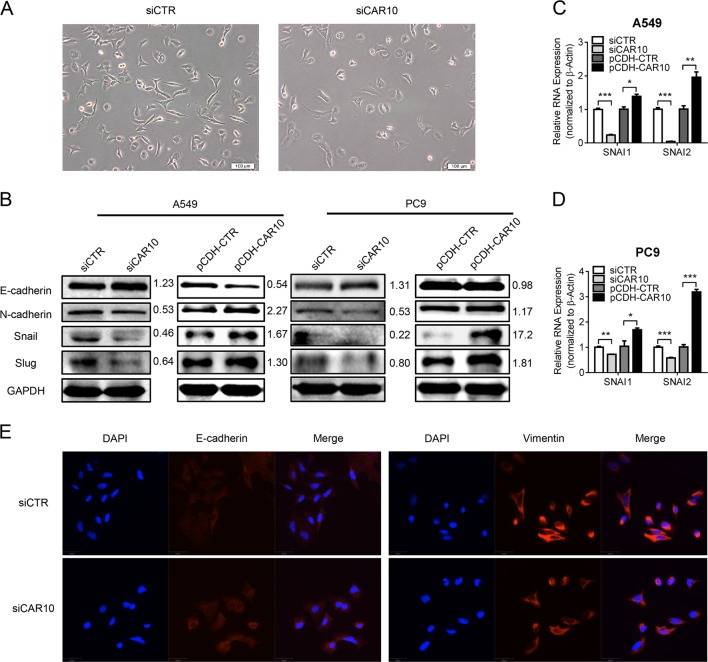
CAR10 induced cell EMT via upregulating *SNAI1* and *SNAI2*. **a** Cell morphology observed by phase-contrast microscopy after A549 cells were transfected with CAR10 Smart Silencer. **b** Western blot analysis of the EMT markers (E-cadherin and N-cadherin) and Snail and Slug expression in A549 and PC9 cells with silencing or overexpressing CAR10. **c**, **d** Quantitative RT-PCR mRNA expression analysis of *SNAI1* and *SNAI2* in A549 and PC9 cells transiently silencing or stably overexpressing CAR10. Data are shown as mean ± SEM; *n* = 3 independent experiments, two-tailed Student’s *t* test. **e** Confocal microscopy images of immunostaining for E-cadherin and vimentin and merged with DAPI in A549 cells with silenced CAR10. Scale bar: 50 μm. *P < 0.05, **P < 0.01, and ***P < 0.001. NS no statistical significance

Compared with A549 control cells’ spike-like shape, we noticed that the CAR10 knockdown cells have cobblestone-like morphology (Fig. 4a). We subsequently studied the correlations between CAR10- and EMT-related genes to further investigate the mechanism via which CAR10 may regulate cell invasion. In both A549 and PC9 cells, the CAR10 knockdown increased the expression of epithelial marker E-cadherin. However, expression of mesenchymal marker N-cadherin as well as transcription factors Snail and Slug decreased in these cells (Fig. 4b). As expected, overexpression of CAR10 decreased E-cadherin expression and increased the expression of N-cadherin, Snail, and Slug (Fig. 4b). Quantitative PCR (qPCR) then detected that CAR10 also upregulated *SNAI1* and *SNAI2* mRNA (Fig. 4c, d). Immunofluorescence analysis also revealed an increase in E-cadherin expression as well as the loss of vimentin expression in CAR10 knockdown A549 cells (Fig. 4e).

To confirm that CAR10 functions in the EMT and metastasis of LUAD, we rescued CAR10 expression in A549 CRISPR-CAR10 cells. As expected, the expression of *SNAI1* and *SNAI2* at both mRNA and protein levels increased when CAR10 expression was rescued (Supplementary Figure 4A and 4B). Transwell and wound-healing assays showed that the regain of CAR10 expression recovered the invasive and migratory abilities of CAR10 knockout cells (Supplementary Figure 4C and 4D). In summary, we revealed that CAR10 is a positive EMT regulator.

### CAR10 stabilized *SNAI1* and *SNAI2* mRNA by sequestering miR-30 and miR-203

**Fig. 5:**
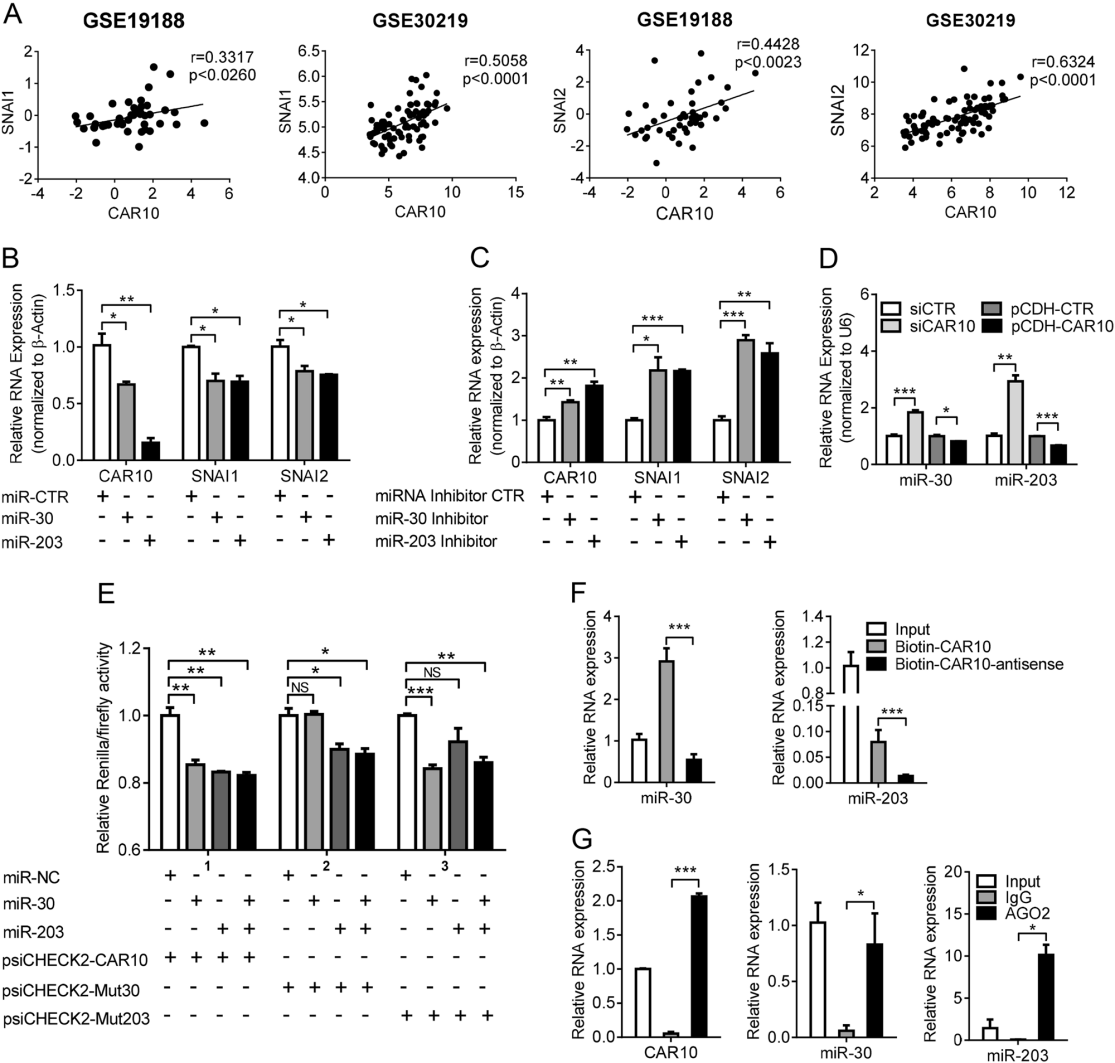
CAR10 sequestered miR-30 and miR-203 to stabilize *SNAI1* and *SNAI2*. **a** *SNAI1* and *SNAI2* expression positively correlated with CAR10 expression in LUAD. Linear regression analysis with the data from datasets GSE19188 and GSE32219 was performed using the Pearson correlation coefficient test. (In linear regression, a Pearson score > 0.3 and *P* < 0.05 were assumed to indicate significance). **b** CAR10 and *SNAI1*/*2* expression levels in A549 cells transfected with miR-30 or miR-203 mimics for 48 h were detected by qRT-PCR. Data are presented as the mean ± SEM of three independent experiments, two-tailed Student’s *t* test. **c** CAR10 and *SNAI1*/*2* expression levels in A549 cells transfected with miR-30 inhibitor or miR-203 inhibitor for 48 h were detected by qRT-PCR. Data are presented as the mean ± SEM of three independent experiments, two-tailed Student’s *t* test. **d** The expression of miR-30 and miR-203 in A549 cells with silenced or overexpressed CAR10 was detected by qRT-PCR. Data are presented as the mean ± SEM of three independent experiments, two-tailed Student’s *t* test. **e** Luciferase activity in A549 cells cotransfected with miR-30 and miR-203 and luciferase reporters containing CAR10 or mutant transcript. Data are presented as the relative ratio of firefly luciferase activity to *Renilla* luciferase activity. Data are shown as mean ± SEM; *n* = 3 independent experiments, two-tailed Student’s *t* test. **f** An RNA pull-down assay followed by biotin-labeled CAR10 and CAR10-antisense to detect miR-30 and/or miR-203 endogenously associated with CAR10. Data are shown as mean ± SEM; *n* = 3 independent experiments, two-tailed Student’s *t* test. **g** AGO2-RIP assay was performed in A549 lysates, followed by qRT-PCR to detect CAR10 and miR-30 or miR-203 associated with AGO2. Data were represented as the mean ± SEM; *n* = 3 independent experiments. *P < 0.05, **P < 0.01, and ***P < 0.001. NS no statistical significance

As in LUAD cells, positive correlations between CAR10 and *SNAI1* as well as *SNAI2* mRNA expression in LUAD tissues were detected in both GSE30219 and GSE19188 GEO datasets (Fig. 5a). Given the location of CAR10 in the cytoplasmic and nuclear fractions, we next evaluated its biological function as a presumptive competing endogenous RNA (ceRNA).

A search of the TargetScan database (http://www.targetscan.org/vert_71/) and miRDB database (http://www.mirdb.org/) indicated that CAR10 contains one region complementary to the “seed” region of miR-30 and two regions complementary to the “seed” region of miR-203 (Supplementary Figure 5A). Both of *SNAI1* and *SNAI2* 3ʹ untranslated regions (UTRs) contained one separate matching region for each of the two miRNAs separately (Supplementary Figure 5A). Not surprisingly, both miR-30 and miR-203 were suppressed in LUAD cells compared with HBE cells (Supplementary Figure 5B). Transfection of miR-30 and miR-203 mimics into A549 and PC9 cells decreased the expression of CAR10, *SNAI1*, and *SNAI2* (Fig. 5b, Supplementary Figure 5C and 5D). Similarly, inhibition of the expression of miR-30 or miR-203 in A549 and PC9 cells increased the expression of CAR10, *SNAI1*, and *SNAI2* (Fig. 5c and Supplementary Figure 5E). Low expression of CAR10 increased the expression of miR-30 and miR-203, and overexpression of CAR10 decreased the expression of miR-30 and/or miR-203 (Fig. 5d and Supplementary Figure 5F).

To determine whether CAR10 is the target of miR-30 or miR-203, we constructed a wild-type and mutated sequence of CAR10 in the 3ʹ UTR of a luciferase CDS (Supplementary Figure 5G) and cotransfected them with miR-30 and/or miR-203 into A549 and PC9 cells. MiR-30 and miR-203 reduced luciferase expression with the wild-type (CAR10), but not the mutated version (Mut30 and Mut203) (Fig. 5e). Similarly, miR-30 and miR-203 decreased reporter activity of WT-*SNAI1*/*2* 3′ UTR, but not the mutant (*SNAI1* and *SNAI2* 3′ UTR Mut) (Supplementary Figure 5H).

**Fig. 6:**
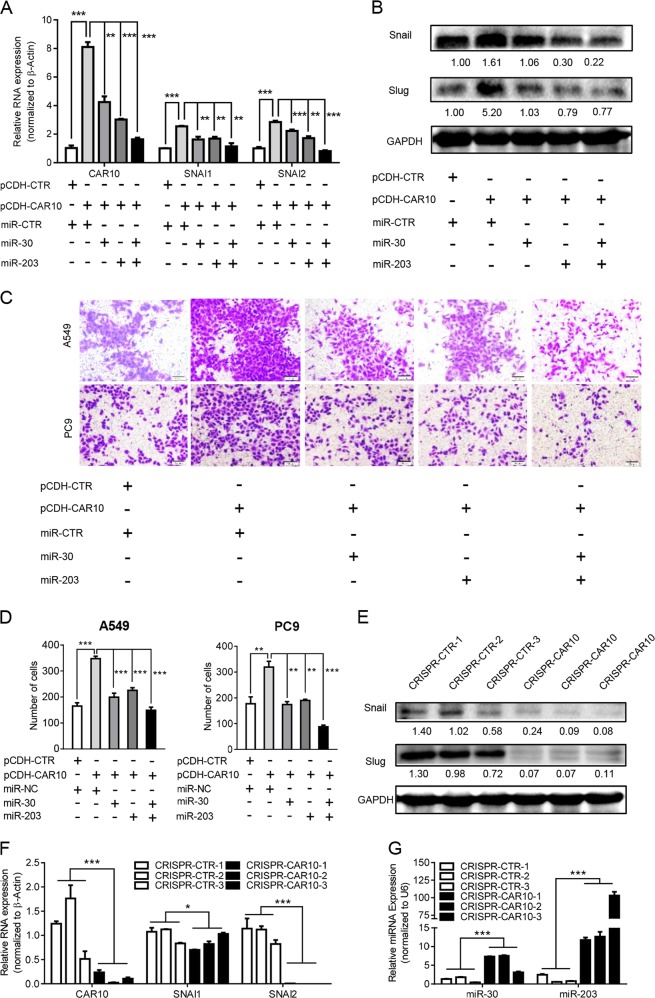
CAR10 promoted EMT through miR-30/203-*SNAI1*/*2* axis. **a**, **b** The mRNA (**a**) or protein levels (**b**) of *SNAI1* and *SNAI2* increased when CAR10 was inhibited by overexpression of miR-30 or/and miR-203 in A549 cells. Data are shown as mean ± SEM; *n* = 3 independent experiments, two-tailed Student’s *t* test. **c**, **d** Overexpression of CAR10 enhanced the invasive potential of A549 and PC9 cells; this change was reversed by co-overexpression of miR-30 or/and miR-203. Data are shown as mean ± SEM; *n* = 3 independent experiments, two-tailed Student’s *t* test. **e** Protein levels of *SNAI1* and *SNAI2* in xenograft tumor tissues of the CRISPR-CTR or CRISPR-CAR10 group detected by western blot assay. **f**, **g** The expression of CAR10, *SNAI1*, *SNAI2*, and miR-30 and miR-203 was monitored by qRT-PCR in CRISPR-CTR or CRISPR-CAR10 xenograft tumor tissue samples. Data are shown as mean ± SEM; n = 3 independent experiments for three CRISPR-CTR tumor tissue samples and three CRISPR-CAR10 tumor tissue samples, two-tailed Student’s *t* test. *P < 0.05, **P < 0.01, and ***P < 0.001. NS no statistical significance

To further investigate the potential direct binding between CAR10 and the miRNAs, we performed an RNA pull-down experiment using biotin-labeled CAR10 and antisense-CAR10 control. Complexes of CAR10 captured on the beads were detected by qPCR. We observed an ~5-fold enrichment of endogenous miR-30 and miR-203 in the CAR10-captured fraction as compared with the control (Fig. 5f). These results indicated that CAR10 binds to miR-30 and miR-203 directly. To determine whether AGO2 is involved in this process, we next performed an RNA immunoprecipitation (RIP) experiment on AGO2. RIP results revealed that CAR10 and miR-30 and miR-203 coprecipitated with AGO2 in A549 cells (Fig. 5g). Moreover, the overexpression of miR-30 or/and miR-203 partly decreased *SNAI1* and *SNAI2* mRNA levels upregulated by the overexpression of CAR10 in A549 cells (Fig. 6a). These pieces of evidence suggested that CAR10 stabilized *SNAI1* and *SNAI2* mRNA via sponging of miR-30 and miR-203.

### CAR10 promoted EMT through miR-30/203-*SNAI1*/*2* axis

Given the role of CAR10 in the regulation of *SNAI1*/*2* expression by sponging miR-30 and/or miR-203, we tested whether CAR10 is involved in EMT through the miR-30/203-*SNAI1*/*2* axis. Upregulation of *SNAI1* and *SNAI2* induced by CAR10 was partly abrogated by miR-30 or miR-203 overexpression (Fig. 6a, b). Simultaneously, the invasion and migration induced by CAR10 could be reversed by miR-30 or miR-203 in LUAD cells (Fig. 6c, d, Supplementary Figure 6A and 6B). Notably, in tumor tissues of the CRISPR-CAR10 nude mice model, the knockout of CAR10 also increased miR-30 and miR-203 expression levels and decreased the mRNA and protein expression levels of *SNAI1* and *SNAI2* (Fig. 6e, f).

We inhibited the expression of miR-30 or/and miR-203 in CAR10 knockout cells. Notably, introduction of miR-30 or miR-203 inhibitors abrogated the inhibitory effect of the CAR10 knockout on the expression of *SNAI1* and *SNAI2* (Supplementary Figure 6C and 6D). Functionally, transwell and wound-healing assays suggested that miR-30 and miR-203 downregulation promoted migration and invasiveness of CAR10 knockout A549 cells (Supplementary Figure 6E). These results indicated that CAR10 promoted EMT and metastasis of LUAD via the miR-30/203-*SNAI1*/*2* axis.

### Diagnostic and prognostic value of CAR10 expression in LUAD patients

**Fig. 7:**
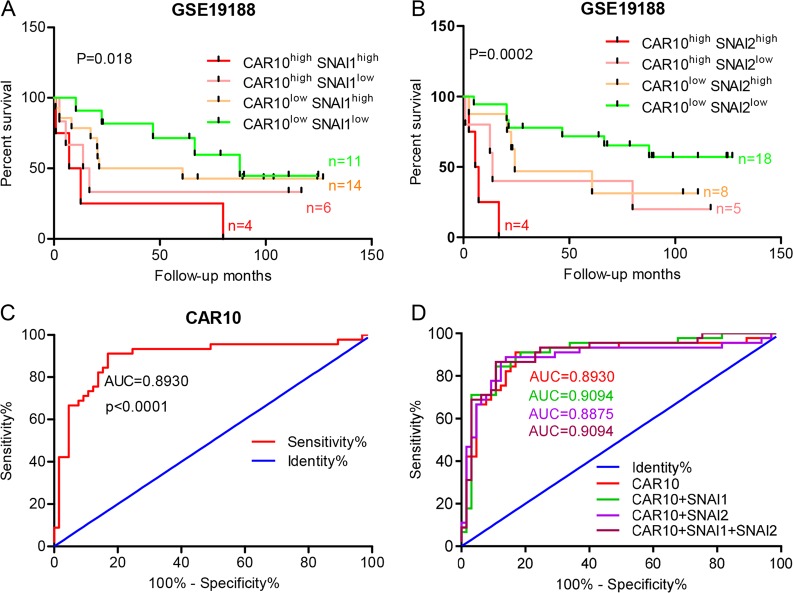
Clinical diagnostic and prognostic value of CAR10 expression in patients with LUAD. **a**, **b** Overall survival (OS) according to Kaplan–Meier analysis shows a difference in the survival between LUAD patients with overexpression of both CAR10 and *SNAI1* or *SNAI2* as compared with the lower expression of both transcripts in the GSE19188 dataset. **c**, **d** Receiver-operating characteristic (ROC) curves displaying the sensitivity and specificity of CAR10 (**c**) or CAR10 combined with *SNAI1*/*2* (**d**) for the diagnosis of LUAD. Insets indicate AUC values, 95% confidence intervals, and statistics

Thus, these results demonstrated that CAR10 can promote LUAD progression in vitro and in vivo. LUAD patients with lower expression of CAR10 in the tumor had longer overall survival (OS) than those with higher CAR10 levels (Fig. 1c, e). We also calculated OS for LUAD patients with LUAD by means of the combined index of CAR10 and *SNAI1*/*2* expression (Fig. 7a, b). We found that LUAD patients tended to have the worst prognosis if the expression of both CAR10 and *SNAI1*/*2* was high, whereas this prognosis was intermediate if only one of the expression variants was upregulated and the other was downregulated. Optimal outcomes were observed if the expression of both variants was low. ROC curve analysis was performed to evaluate the diagnostic values of CAR10 for LUAD. As depicted in Fig. 7c, the area under the curve of CAR10 for the diagnosis was 0.8930 with a 95% confidence interval of 0.8241–0.9619 (*P* < 0.0001). We also analyzed the ROC curve of CAR10 combined with *SNAI1*/*2*, but the assessment of efficiency uncovered no further improvement (Fig. 7d). These results suggested that the expression of CAR10 in LUAD tissues might be a promising prognostic and diagnostic indicator.

## Discussion

LncRNAs perform important functions in carcinogenesis and cancer progression. In this study, we screened lncRNAs differentially expressed between tumor tissues and matched nontumorous lung tissues from patients with metastatic LUAD by human transcriptome microarray analysis. We found lncRNA CAR10 to be upregulated in tumor tissues and even more overexpressed in paired metastatic tissues of LUAD. Elevated expression of CAR10 was found to correlate positively with the poor prognosis of LUAD. Besides, CAR10 increased the proliferation of lung cancer cells as previously reported [16]. Moreover, we demonstrated that CAR10 promotes migration and invasiveness abilities of LUAD cells and a knockout of CAR10 by means of the CRISPR/Cas9 system inhibited the metastasis of cancer cells in nude mice. Indeed, silencing of CAR10 induced epithelial-like morphological features in A549 cells and downregulated the mesenchymal marker N-cadherin and upregulated the epithelial marker E-cadherin. CAR10 also led to an increase of Snail and Slug expression (two master transcription factors of EMT) and enhanced the EMT process in LUAD cells.

EMT is a reversible process by which a polarized epithelial cell is transformed into a cell with a mesenchymal phenotype [19]. Metastasis is the leading cause of cancer-related deaths, and EMT is thought to be important for metastasis [20]. Until recently, few publications have emphasized the role of lncRNAs in EMT and tumor metastasis [21–23]. MALAT1 has been found to be upregulated in lung cancer [24] and alters the expression of EMT-associated genes, thus promoting brain metastasis [10]. HOTAIR is required for tumor metastasis because of its key function in different signaling mechanisms related to EMT [25]. We found that CAR10 significantly promotes EMT, and its expression level correlates with *SNAI1* and *SNAI2* expression in LUAD cells and tissues. Herein, this is the first demonstration that CAR10 may act as an miRNA sponge to regulate *SNAI1* and *SNAI2* expression. We found that CAR10 shares miR-30 and miR-203 response elements with *SNAI1* and *SNAI2*. Unsurprisingly, CAR10 was found to be negatively regulated by miR-30 and miR-203 in LUAD cells. A luciferase assay confirmed that miR-30 and miR-203 target CAR10. CAR10 can directly bind miR-30 and miR-203, and AGO2 takes part in this process. According to the existing literature [26–28], miR-30 and miR-203 indeed target *SNAI1* and *SNAI2*, in agreement with our results. The upregulation of *SNAI1* and *SNAI2* and the increasing invasive and migratory abilities induced by CAR10 were attenuated by miR-30 or miR-203 overexpression. Furthermore, CAR10 knockout successfully decreased the expression of *SNAI1* and *SNAI2* and inhibited tumor proliferation and metastasis in vivo. These results support the conclusion that CAR10 directly binds to miR-30 and miR-203 to reduce their inhibitory effect on *SNAI1* and *SNAI2*, thus contributing to the metastasis of LUAD.

Lately, a set of genes, including ncRNAs and coding genes, have been shown to correlate with prognosis of LUAD patients [23, 29–33]. The increased CAR10 expression was found to significantly correlate with the poor prognosis of LUAD patients, and the expression of CAR10 compared with *SNAI1* and *SNAI2* as multi-indexes may more effectively evaluate the prognosis of LUAD patients. Furthermore, CAR10 was found to have good efficiency of diagnosis of LUAD patients, and this efficiency was not improved when combined with *SNAI1* or *SNAI2*. Therefore, CAR10 has the potential to become a diagnostic and prognostic marker of LUAD. However, the exact applicable cutoff value of CAR10 still needs to be confirmed in a large set of patients. In addition, some of the ncRNAs that are stably expressed in plasma, especially miRNAs may serve as the biomarkers for LUAD diagnosis and treatment [34–36]. Whether CAR10 can also be detected in the plasma from LUAD patients and whether the circulating CAR10 is also associated with LUAD development are important and interesting questions for further investigation.

In conclusion, lncRNA CAR10 was demonstrated to be an oncogene that promotes proliferation, migration, and invasiveness of LUAD cells. Additionally, overexpression of CAR10 is related to an adverse prognosis for LUAD patients. As a tumor metastasis regulator, CAR10 enhances the expression of *SNAI1* and *SNAI2* via directly sponging miR-30 and miR-203 to promote EMT. CAR10 is likely to have a significant potential as a diagnostic and prognostic marker and a therapeutic target in LUAD if the interaction of CAR10 with miR-30/203 and *SNAI1*/*2* is blocked.

## Materials and methods

### Cell culture

NCI-H358, A549, PC9, B16, AGS, MCF7, HeLa, HepG2, SW480, BESA-2B cells, HEK293, HEK293T, and the normal human bronchial epithelial cell line (HBE) were obtained from the cell bank of Cancer Research Institute of Central South University (Hunan, China) and were tested using RT–PCR for mycoplasma contamination. The cell lines have been purchased from ATCC and authenticated by STR profiling, so authentications were not performed. NCI-H358, A549, PC9, B16, AGS, MCF7, HeLa, HepG2, SW480, BESA-2B, and HBE cells were cultured in RPMI-1640 (Gibco, Life Technology, Carlsbad, CA, USA). HEK293 and HEK293T cells were grown in DMEM medium (Gibco, Life Technology, Carlsbad, CA, USA). Both RPMI-1640 and DMEM medium were supplemented with 10% FBS and 1% penicillin/streptomycin and all cell lines were grown at 37 °C and 5% CO_2_.

### Clinical specimens

Set1: Three pairs of lung adenocarcinoma (LUAD) samples were collected in this study, which included LUAD tissue samples and matched para-carcinoma tissue samples, respectively, coming from three patients for HTA 2.0 microarray analysis. These three pairs samples were collected from diagnosed LUAD patients with lymph node metastasis at the Hunan Province Tumor Hospital (Changsha, China). SET2: In total, 74 pairs of LUAD samples were collected to analyze the expression of CAR10 in tumor tissue samples compared with matched para-carcinoma tissues. These 74 pairs samples were collected from diagnosed LUAD patients, including 37 patients with lymph node metastasis and 37 patients without lymph node metastasis at the Second Xiangya Hospital (Changsha, China). The patients were informed regarding the sample collection and signed informed consented forms. Collection and use of tissue samples were approved by the Ethical Review Committee of Hunan Province Tumor Hospital and the Second Xiangya Hospital. Clinic information was collected from patient medical records and are reported in Supplementary Table 1.

### Human transcriptome microarray

The goal of this study was to identify lncRNAs upregulated in LUAD that contributed to the metastasis of LUAD and positively related to poor prognosis. Total RNA was extracted from three LUAD tissue samples and three matched normal adjacent lung tissues using the RNeasy mini kit (Qiagen, Valencia, CA, USA) and quantified using Nanodrop ND-100 Spectrophotometer (Thermo Fisher Scientific, USA). RNA samples were then subjected to RNA amplification using the Sensation Plus FFPE Amplification and WT Labeling Kit (Affymetrix Inc., Santa Clara, CA, USA), as previously reported [37, 38]. The biotin double-stranded cDNA products were hybridized to Affymetrix HTA 2.0 arrays using an Affymetrix hybridization kit. Hybridized HTA 2.0 arrays were scanned with an Affymetrix GeneChip^®^ 3000 fluorescent scanner. Image generation and feature extraction was performed using Affymetrix GeneChip Command Console Software. The raw data (.*CEL) were analyzed using the Transcriptome Analysis Console (TAC) 4.0 software, which allows for the identification of differentially expressed genes (DEG) and exons and the visualization of alternative splicing events for determining possible transcript isoforms that may exist in samples.

To explore CAR10 expression in other databases, we downloaded Gene Expression Omnibus (GEO) (http://www.ncbi.nlm.nih.gov/geo/) datasets GSE19188 (including 45 LUAD tissue samples and 65 normal lung tissue samples) and GSE30219 (including 85 LUAD tissue samples and 14 normal lung tissue samples). These two microarray (based on Affymetrix U133 Plus 2.0) gene expression included 3053 lncRNAs [39], we reanalyzed the expression of CAR10 between LUAD tissue samples and normal lung tissue samples. The Kaplan–Meier survival analysis of CAR10 in LUAD tissue samples was executed in GEO datasets GSE19188 and GSE30219.

### Antibodies and reagents

Information on antibodies used in this study is provided in Supplementary Table 4. The EMT-related genes of antibodies against ZO-1, ZEB1, N-cadherin, E-cadherin, β-catenin, vimentin, Snail, Slug, and claudin-1 were used to test the EMT markers of cells in the study purchased from Cell Signaling Technology, Inc. (USA). MiR-30/203 mimics, miR-30/203 inhibitors, and forward primers were purchased from RiboBio Co. (Guangzhou, China). Primers used are listed in Supplementary Table 5.

### Plasmid construction and cell transfection

To construct a plasmid expressing CAR10, the full-length human CAR10 sequence (NCBI: Homo sapiens cDNA FLJ31066) was synthesized and subcloned into the pcDNA3.1 vector (Sangon, Shanghai, China). The plasmids were transfected into cells using Lipofectamine3000 Reagent (Invitrogen^**TM**^, USA). In order to establish the stably CAR10-overexpressing cells, CAR10 sequence was subcloned into the pCDH vector. For knockdown of CAR10, lncRNA Smart Silencer (RiboBio, China), a mixture of three siRNAs and three antisense oligonucleotides (ASOs) was transfected into cells with Lipofectamine3000 (Invitrogen^**TM**^). CAR10 siRNAs and lncRNA Smart Silencer sequences are shown in Supplementary Table 6.

### RNA isolation and real-time PCR analysis

Total RNA was extracted using Trizol reagent (CWIO, Beijing, China) according to the manufacturer’s protocol. The RNA quantity and quality were evaluated by Nanodrop ND-2000 spectrophotometer (Thermo Scientific™, USA). For mRNA and lncRNA expression analysis, 2 μg of total RNA was reverse transcribed into cDNA with RevertAid First Strand cDNA Synthesis Kit (Thermo Scientific™, USA). For miRNA expression analysis, 2 μg of total RNA was reverse transcribed into cDNA with miDETECT A Track™ qRT-PCR Kit (Guangzhou RiboBio Co., China). qRT-PCR was performed with 2x SYBR Green qPCR Master Mix (Bimake, China) in Bio-Rad CFX Connect™ Real-Time PCR Detection System (Bio-Rad, USA). The primers used were as follows: ACTB, GAPDH, or U6 were used as the reference and normalization control. The average of three independent analyses for each gene was calculated. The 2^–ΔΔCt^ method was used to determine the relative quantification of gene expression levels.

### CCK8 assay

Cells were transfected with siCAR10 or scrambled siCTR (NC) for 48 h, and then seeded in a 96-well plate at a density of 1000 cells/well. The stably CAR10 over-expressing cell lines of A549 and PC9 were cultured to logarithmic growth phase, and then seeded in a 96-well plate at a density of 800 cells/well. Changes in cell viability were determined by adding 20 μL of CCK8 solution (5 mg/ml) to each well at 0, 1, 2, 3, 4, or 5-day time points. The plate was incubated at 37 °C for an additional 2 h. The optical density of each well was determined with a scanning multi-well spectrophotometer at a wavelength of 450 nm. The experiments were repeated three times and six parallel samples were measured each time.

### Colony formation assay

Cells transfected with LncRNA Smart Silencer targeting CAR10 for 48 h or stable overexpression of CAR10 cells were trypsinized into a single-cell suspension. About 1000 cells were plated in each well of the six-well plate and maintained for 2 weeks to form a colony. Then the plates were gently washed with PBS and immobilized the cells with 1% formaldehyde solution, and then stained with 0.1% of crystal violet. Colonies with over 50 cells were manually counted. The number of colonies was calculated by Image-Pro Plus 6.0.

### Wound-healing assay

To investigate the difference of the cell migration capability between up- or low expression of CAR10 and negative treatment cells, a modified wound-healing assay was performed, as previously reported [40]. When cells were grown to confluent monolayers, four wounds were created using a sterile 10-μL pipette tip followed by washing with D-Hanks to remove detached cells. Cells were then cultured in a medium with 2% serum. Images were captured at 0, 24, 48, and 72 h after wounding using a microscope (Nikon).

### Transwell invasion assay

Boyden chamber invasion assay (24-well plate format) was used to investigate the ability of cell invasion. Corning Costar Transwell 24-well plates (8-μm pores; Corning, USA) were coated with Matrigel (BD) and placed in a cell culture hood for 3 h at 37 °C. Then a total of 20,000 cells/well were seeded in the inserts and cultured in the medium without serum. Cultured medium with 20% serum was placed in the bottom wells. Cells were then allowed to invade for 48 h. Invaded cells were fixed with 1% formaldehyde solution for 15 min and stained with 0.1% of crystal violet for 15 min. Then, a cotton swab was used to erase the noninvasive cells and Matrigel on the top surface of the membrane. Invasive cells on the lower surface of the membrane were stained by crystal violet in purple and the stained cells were captured using a microscope (Nikon) and counted by software Image-Pro Plus 6.0.

### Western blotting assay

The protein of cells and tissue samples was extracted using radioimmunoprecipitation assay buffer (RIPA buffer, Beyotime Biotechnology, Haimen, China). The concentration of protein was quantified using the Pierce™ BCA Protein Assay Kit (Thermo Scientific™, USA). Equal amounts of protein (60 μg) were separated by 10% sodium dodecyl sulfate-polyacrylamide gel electrophoresis and transferred to a PVDF membrane (Millipore). After being blocked with 5% nonfat milk in TBS Tween 20 (TBST; 25 mM Tris, pH 7.5, 150 mM NaCl, and 0.1% Tween 20) for 1 h at room temperature, membranes were incubated with primary specific antibodies in 5% bovine albumin (BSA) in TBST overnight at 4 °C. After washing three times with TBST, membranes were then incubated with horseradish peroxidase-labeled secondary antibody for 1 h at 37 ℃. The signal was visualized using an ECL detection reagent (BioRad, USA) and quantified by densitometry (BioRad ChemiDoc XRS system).

### Dual-luciferase reporter assay

Dual-Luciferase^®^ Reporter Assay System was performed according to the manufacturer’s instructions (Promega). To evaluate the interaction between miR-30/203 and CAR10, miR-30/203 and *SNAI1*/*2* 3ʹUTR cells were transfected with psiCHECK2-based constructs containing CAR10, CAR10-Mut30, CAR10-Mut203, *SNAI1* 3′ UTR, *SNAI1* 3ʹ UTR-Mut, *SNAI2* 3′UTR and *SNAI2* 3′ UTR-Mut plus miR-30, or/and miR-203 mimics. Forty-eight hours later, firefly and *Renilla* luciferase activity was examined by the Dual-Luciferase Reporter Assay System, and Renilla activity was used to normalize firefly activity.

### RNA immunoprecipitation (RIP) assay

To determine whether CAR10 and miR30/203 were associated with the RNA-induced silencing complex (RISC), RIP experiment was performed with the AGO2 antibody (ab32381, Abcam, USA) following the manufacturer’s protocol. qRT-PCR analysis was performed to measure the expression levels of CAR10 and miR-30/203. Normal mouse IgG (Millipore, USA) was used as a negative control.

### Biotin pull-down assay

LncRNA-CAR10 and CAR10-antisense (negative control) were in vitro transcribed, respectively, from vector pcDNA3.1-CAR10 and pcDNA3.1-CAR10-antisense, and biotin-labeled with the Biotin RNA Labeling Mix (Roche, Mannheim, Germany) and T7 RNA polymerase (Roche), treated with RNase-free DNase I (Takara, Japan). The biotin-labeled RNA was purified with the RNeasy Mini Kit (Qiagen). Cell lysates from A549 cells (1 × 10^7^) were incubated with 2 μg of purified biotin-labeled transcripts for 1 h at 25 °C. The biotin-labeled RNA was isolated with Dynabeads™ M-270 Streptavidin (Invitrogen^TM^). The CAR10 and miR-30/203 present in the pull-down complexes were detected by qRT-PCR analysis.

### CAR10 KO by CRISPR/Cas9

Selection of CAR10 KO clones in A549 cells was carried out using the procedure as described previously [41]. Dual sgRNAs of CAR10 were researches in Zhang Lab website (http://crispr.mit.edu/). Then, sgRNAs were designed in two donor vectors (lentiCRISPR-v2 and pLKO5.sgRNA.EFS.tRFP580) that were cotransfected with pMD.2 G and psPAX2 into the HEK293T cells in a 6-mm dish using Neofect™ DNA transfection reagent (Neofect (Beijing) biotech, China). Forty-eight hours later after transfection, a supernatant from lentivirus-producing cells filtered through 0.45-μm filters was added to six-well plates with A549 cells. Positively infected cells were isolated using puromycin and flow cytometry. CAR10 deletion from each single clone was then confirmed by PCR with specific primers. Oligonucleotide sequences of sgRNAs and primers used to choose CRISPR-CAR10 A549 cells are shown in Supplementary Table 7.

### Animal experiments

To confirm the role of CAR10 in the promotion of LUAD cell proliferation in vivo, we performed subcutaneous tumor mouse models. Twenty female BALB/c nude mice (4-week-old) were randomly divided into two groups, with ten mice in each group. The CAR10 KO and negative control cells (1 × 10^6^) were washed once with PBS and subcutaneously injected into nude mice (*n* = 10). The tumors were measured every 2 days 3 weeks after injection, and the growth curves were plotted for each group. The tumor volumes were estimated using the following formula: Volume = length × width^2^ × 0.52. All mice were killed 36 days after inoculation, and tumors were isolated and photographed. All photographed tumors were isolated from single experiments at a similar time point. Part of the subcutaneous tumors were fixed in 10% formalin and embedded in paraffin for IHC and ISH. The rest of tumors were stored in liquid nitrogen for detecting the expression of CAR10, *SNAI1*, *SNAI2*, miR-30, and miR-203 by western blotting or qRT-PCR in this study, and at least three mice in each group were analyzed in at least three separate experiments.

To further confirm the pro-metastatic potential of CAR10 in the positive impact on LUAD cells metastasis in vivo, we performed metastatic tumor mouse models. Twenty female BALB/c nude mice (4-week-old) were randomly divided into two groups. CAR10 KO or negative control A549 cells were transfected with pLenti6 V5 D-TOPO Luciferase EGFP (CMV/Luciferase17-EGFP&BSD) vector and were injected into the tail veins of the nude mice (4 × 10^6^ cells per mice). Optical in vivo imaging of cancer metastasis was monitored with in vivo luminescence imaging system (IVIS) at 7 and 8 weeks after injection. The mice were killed 8 weeks after injection and the lungs and lymph nodes were removed for further analysis. All animals in the study were supplied by Hunan SJA Laboratory Animal Company (Hunan, China). Animal experiments were approved by the Institutional Animal Care and Use Committee of Central South University (Changsha, China).

### Statistical analysis

Statistical analysis was performed using SPSS software, version 20.0 (SPSS, IBM, USA) and GraphPad Prism (version 5.01, La Jolla, CA, USA) software. Student’s *t* tests were used to evaluate significant differences between any two groups of data and one-way ANOVA was used to evaluate significant differences for multiple comparisons. Overall survival (OS) was calculated using the Kaplan–Meier method, and the results of the analysis were considered significant in a log-rank test if *P* < 0.05. All data are represented as means ± standard deviation. Differences were considered significant if *P* < 0.05. *P < 0.05; **P < 0.01; ***P < 0.001.

## Supplementary information

Supplementary Figure caption

Supplementary Figure 1

Supplementary Figure 2

Supplementary Figure 3

Supplementary Figure 4

Supplementary Figure 5

Supplementary Figure 6A-B

Supplementary Figure 6C-E

Supplementary Table 1

Supplementary Table 2

Supplementary Table 3

Supplementary Table 4

Supplementary Table 5

Supplementary Table 6

Supplementary Table 7

## References

[1] Chen W, Zheng R, Baade PD, Zhang S, Zeng H, Bray F (2016). Cancer statistics in China, 2015. CA Cancer J Clin.

[2] Siegel RL, Miller KD, Jemal A (2017). Cancer statistics, 2017. CA Cancer J Clin.

[3] Travis WD (2014). The 2015 WHO classification of lung tumors. Pathologe.

[4] Paez JG, Janne PA, Lee JC, Tracy S, Greulich H, Gabriel S (2004). EGFR mutations in lung cancer: correlation with clinical response to gefitinib therapy. Science.

[5] Soda M, Choi YL, Enomoto M, Takada S, Yamashita Y, Ishikawa S (2007). Identification of the transforming EML4-ALK fusion gene in non-small-cell lung cancer. Nature.

[6] Jen J, Tang Y, Lu Y, Lin C, Lai W, Wang Y (2017). Oct4 transcriptionally regulates the expression of long non-coding RNAs NEAT1 and MALAT1 to promote lung cancer progression. Mol Cancer.

[7] Xiang Y, Zhang Y, Tang Y, Li Q (2017). MALAT1 modulates TGF-β1-induced endothelial-to-mesenchymal transition through downregulation of miR-145. Cell Physiol Biochem.

[8] Ji P, Diederichs S, Wang W, Boing S, Metzger R, Schneider PM (2003). MALAT-1, a novel noncoding RNA, and thymosin beta4 predict metastasis and survival in early-stage non-small cell lung cancer. Oncogene.

[9] Liu M, Sun W, Liu Y, Dong X (2016). The role of lncRNA MALAT1 in bone metastasis in patients with non-small cell lung cancer. Oncol Rep.

[10] Shen L, Chen L, Wang Y, Jiang X, Xia H, Zhuang Z (2015). Long noncoding RNA MALAT1 promotes brain metastasis by inducing epithelial-mesenchymal transition in lung cancer. J Neurooncol.

[11] Lin L, Gu ZT, Chen WH, Cao KJ (2015). Increased expression of the long non-coding RNA ANRIL promotes lung cancer cell metastasis and correlates with poor prognosis. Diagn Pathol.

[12] Pasmant E, Laurendeau I, Heron D, Vidaud M, Vidaud D, Bieche I (2007). Characterization of a germ-line deletion, including the entire INK4/ARF locus, in a melanoma-neural system tumor family: identification of ANRIL, an antisense noncoding RNA whose expression coclusters with ARF. Cancer Res.

[13] Nie FQ, Sun M, Yang JS, Xie M, Xu TP, Xia R (2015). Long noncoding RNA ANRIL promotes non-small cell lung cancer cell proliferation and inhibits apoptosis by silencing KLF2 and P21 expression. Mol Cancer Ther.

[14] Mondal T, Rasmussen M, Pandey G, Isaksson A, Kanduri C (2010). Characterization of the RNA content of chromatin. Genome Res.

[15] Guo H, Zhang X, Dong R, Liu X, Li Y, Lu S (2014). Integrated analysis of long noncoding RNAs and mRNAs reveals their potential roles in the pathogenesis of uterine leiomyomas. Oncotarget.

[16] Wei M, Zhou Y, Wen Z, Zhou B, Huang Y, Wang G (2016). Long non-coding RNA stabilizes the Y-box-binding protein 1 and regulates the epidermal growth factor receptor to promote lung carcinogenesis. Oncotarget.

[17] Hou J, Aerts J, den Hamer B, van Ijcken W, den Bakker M, Riegman P (2010). Gene expression-based classification of non-small cell lung carcinomas and survival prediction. PLoS ONE.

[18] Rousseaux S, Debernardi A, Jacquiau B, Vitte AL, Vesin A, Nagy-Mignotte H (2013). Ectopic activation of germline and placental genes identifies aggressive metastasis-prone lung cancers. Sci Transl Med.

[19] Otsuki Y, Saya H, Arima Y (2018). Prospects for new lung cancer treatments that target EMT signaling. Dev Dyn.

[20] Savagner P (2010). The epithelial-mesenchymal transition (EMT) phenomenon. Ann Oncol.

[21] Klingenberg M, Gross M, Goyal A, Polycarpou-Schwarz M, Miersch T, Ernst AS (2018). The lncRNA CASC9 and RNA binding protein HNRNPL form a complex and co-regulate genes linked to AKT signaling. Hepatology.

[22] Schmitt AM, Chang HY (2016). Long noncoding RNAs in cancer pathways. Cancer Cell.

[23] Yuan S, Liu Q, Hu Z, Zhou Z, Wang G, Li C (2018). Long non-coding RNA MUC5B-AS1 promotes metastasis through mutually regulating MUC5B expression in lung adenocarcinoma. Cell Death Dis.

[24] Li CH, Chen Y (2013). Targeting long non-coding RNAs in cancers: progress and prospects. Int J Biochem Cell Biol.

[25] Padua Alves C, Fonseca AS, Muys BR, de Barros ELBR BurgerMC, de Souza JE (2013). Brief report: the lincRNA Hotair is required for epithelial-to-mesenchymal transition and stemness maintenance of cancer cell lines. Stem Cells.

[26] Huang J, Yao X, Zhang J, Dong B, Chen Q, Xue W (2013). Hypoxia-induced downregulation of miR-30c promotes epithelial-mesenchymal transition in human renal cell carcinoma. Cancer Sci.

[27] Kao CJ, Martiniez A, Shi XB, Yang J, Evans CP, Dobi A (2014). miR-30 as a tumor suppressor connects EGF/Src signal to ERG and EMT. Oncogene.

[28] Zhang Z, Zhang B, Li W, Fu L, Fu L, Zhu Z (2011). Epigenetic silencing of miR-203 upregulates SNAI2 and contributes to the invasiveness of malignant breast cancer cells. Genes Cancer.

[29] Wang D, Gao Z, Han L, Xu F, Liu K, Shen Y (2017). Long noncoding RNA CASC2 inhibits metastasis and epithelial to mesenchymal transition of lung adenocarcinoma via suppressing SOX4. Eur Rev Med Pharmacol Sci.

[30] Wang Q, Cheng N, Li X, Pan H, Li C, Ren S (2017). Correlation of long non-coding RNA H19 expression with cisplatin-resistance and clinical outcome in lung adenocarcinoma. Oncotarget.

[31] Wei Y, Yan Z, Wu C, Zhang Q, Zhu Y, Li K (2017). Integrated analysis of dosage effect lncRNAs in lung adenocarcinoma based on comprehensive network. Oncotarget.

[32] Wen X, Han X, Wang Y, Fan S, Zhuang J, Zhang Z (2018). Effects of long noncoding RNA SPRY4-IT1-mediated EZH2 on the invasion and migration of lung adenocarcinoma.. J Cell Biochem.

[33] Zhou M, Xu W, Yue X, Zhao H, Wang Z, Shi H (2016). Relapse-related long non-coding RNA signature to improve prognosis prediction of lung adenocarcinoma. Oncotarget.

[34] Jiang S, Wang R, Yan H, Jin L, Dou X, Chen D (2016). MicroRNA-21 modulates radiation resistance through upregulation of hypoxia-inducible factor-1alpha-promoted glycolysis in non-small cell lung cancer cells. Mol Med Rep.

[35] Liu H, Zhou G, Fu X, Cui H, Pu G, Xiao Y (2017). Long noncoding RNA TUG1 is a diagnostic factor in lung adenocarcinoma and suppresses apoptosis via epigenetic silencing of BAX. Oncotarget.

[36] Xu S, Yang F, Liu R, Li X, Fan H, Liu J (2018). Serum microRNA-139-5p is downregulated in lung cancer patients with lytic bone metastasis. Oncol Rep.

[37] Pillai R, Deeter R, Rigl CT, Nystrom JS, Miller MH, Buturovic L (2011). Validation and reproducibility of a microarray-based gene expression test for tumor identification in formalin-fixed, paraffin-embedded specimens. J Mol Diagn.

[38] Roberts L, Bowers J, Sensinger K, Lisowski A, Getts R, Anderson MG (2009). Identification of methods for use of formalin-fixed, paraffin-embedded tissue samples in RNA expression profiling. Genomics.

[39] Van Grembergen O, Bizet M, de Bony EJ, Calonne E, Putmans P, Brohee S (2016). Portraying breast cancers with long noncoding RNAs. Sci Adv.

[40] Rodriguez LG, Wu X, Guan JL (2005). Wound-healing assay. Methods Mol Biol.

[41] Ho TT, Zhou N, Huang J, Koirala P, Xu M, Fung R (2015). Targeting non-coding RNAs with the CRISPR/Cas9 system in human cell lines. Nucleic Acids Res.

