# Supplementary material for: Long noncoding RNA CAR10 promotes lung adenocarcinoma metastasis via miR-203/30/SNAI axis
Source: Oncogene. 2019 Jan 7;38(16):3061–76. doi: 10.1038/s41388-018-0645-x (PMC6484688; doi:10.1038/s41388-018-0645-x)
Supplement: Supplementary file 1 — Supplementary Figure caption [file 41388_2018_645_MOESM1_ESM.docx]

**Supplementary Figure 1.** Identification of differentially expressed lncRNA candidates in lung adenocarcinoma.

(**A**) The flow chart of selected differential expression between tumor and para-tumor samples. (**B**) Volcano plots revealed differentially expressed lncRNAs between LUAD tissues and matched non-tumor lung tissues in human transcriptome microarray (Affymetrix HTA 2.0) analysis. Cutoff criteria were an absolute fold change of ≥1.5 and *p* < 0.05. (**C**) The basic expression of six upregulated lncRNA candidates in the lung tissue according to NONCODE RNA sequence databases. (**D**) CAR10 expression was analyzed by qRT-PCR in matched tumor (n = 4) and metastatic tissue samples (n = 4). All data are presented as the mean ± SEM of three independent experiments, where appropriate, and were analyzed using pair two-tailed Student’s *t*-test. **p* <0.05, ** *p* <0.01, *** *p* <0.001; NS: no statistical significance.

**Supplementary Figure 2.** CAR10 regulated the proliferation of LUAD cells *in vitro*.

(**A**) Relative expression of CAR10 in total RNA, cytoplasmic RNA and nuclear RNA samples from A549 cells transfected with lncRNA Smart Silencer or normal siRNAs targeting CAR10 as compared to negative control. Data are presented as the mean ± SEM of three independent experiments, two-tailed Student’s *t*-test. (**B, C**) The CCK8 assay was performed on A549 and PC9 cells transfected with CAR10 lncRNA Smart Silencer or stably CAR10 overexpressing cells. n = 3, non-parametric Mann-Whitney test. (**D, E**) Silencing CAR10 and overexpression of CAR10 regulated colony formation by cells as indicated. Number of colonies was shown as mean ± SEM; n = 3 independent experiments, two-tailed Student’s *t*-test. **p* <0.05, ** *p* <0.01, *** *p* <0.001, NS: no statistical significance.

**Supplementary Figure 3.** CAR10 silencing inhibits tumor growth and metastasis *in vivo*.

(**A**) sgRNA sequences targeting total CAR10 transcript were designed on the CRISPR DESIGN website <http://crispr.mit.edu/>, and the primers were designed to screen cell colonies with a knockout of CAR10. Primers are shown in Supplementary Table 6. (**B**) PCR analysis of the targeted locus showing the band corresponding to the genomic deletion in cell colonies, and a knockout-positive colony of A549 cells was chosen for further research. Every colony was detected two times. (**C**) qRT-PCR analysis of CAR10 expression in A549 cells treated with CRISPR/Cas9 system. Data are shown as mean ± SEM; n = 3 independent experiments, two-tailed Student’s *t*-test. (**D**) Invasive capacities of control (CRISPR-CTR) or CAR10 knocked out (CRISPR-CAR10) A549 cells were monitored in a Transwell assay. Data are shown as mean ± SEM; n = 3 independent experiments, two-tailed Student’s *t*-test. (**E**) Tumor-bearing mice were photographed with inhalational anesthesia, and the arrow indicates the tumor burden. **p* < 0.05, ***p* < 0.01, and ****p* < 0.001, NS: no statistical significance.

**Supplementary Figure 4.** Re-expression of CAR10 rescued migration and invasiveness abilities of CRISPR-CAR10 A549 cells.

(**A**) Quantitative RT-PCR mRNA expression analysis of CAR10, *SNAI1* and *SNAI2* after CAR10 was re-expressed in CRISPR-CAR10 A549 cells. Data are shown as mean ± SEM; n = 3 independent experiments, two-tailed Student’s *t*-test. (**B**) The Snail and Slug protein expression in CRISPR-CAR10 A549 cells with or without overexpression of CAR10. (**C, D**) pcDNA3.1-CAR10 and control plasmids were transfected into CRISPR-CAR10 A549 cells, and cell invasion was assessed by a Transwell assay, whereas cell migration was analyzed by a wound-healing assay. Data are shown as mean ± SEM; n = 3 independent experiments, two-tailed Student’s *t*-test. **p* <0.05, ***p* <0.01, ****p* <0.001, NS: no statistical significance.

**Supplementary Figure 5.** CAR10 as sponge interacted with miR-30 and miR-203 to regulate *SNAI1* and *SNAI2* expression.

(**A**) Binding sites of miR-30 and miR-203 in the CAR10 transcript and *SNAI1*/*2* 3′UTR were predicted based on TargetScan and miRDB databases. The red nucleotides are the seed sequences of microRNAs. (**B**) qRT-PCR analysis of the expression of miR-30 and miR-203 in four LUAD cell lines (A549, PC9, Calu3 and H1975) and two immortalized normal cell lines (HBE and HEK293). Data are presented as the mean ± SEM of three independent experiments, two-tailed Student’s *t*-test. (**C, D**) miR-30 and/or miR-203 expression in A549 and PC9 cells transfected with miR-30 mimics, miR-203 mimics, or negative control oligonucleotides (miR-CTR). Data are presented as the mean ± SEM of three independent experiments, two-tailed Student’s *t*-test. (**E**) miR-30/203 expression in A549 and PC9 cells transfected with a miR-30 inhibitor or miR-203 inhibitor or negative control oligonucleotides (miR-inhibitor CTR). Data are presented as the mean ± SEM of three independent experiments, two-tailed Student’s *t*-test. (**F**) The expression of miR-30 and miR-203 in PC9 cells was detected by qRT-PCR after silencing or overexpressing CAR10. Data are presented as the mean ± SEM of three independent experiments, two-tailed Student’s *t*-test. (**G**) Schematic illustration of psiCHECK2-based luciferase reporter constructs containing: wild-type CAR10 (psiCHECK2-CAR10), wild-type *SNAI1* 3′UTR and wild-type *SNAI2* 3′UTR; mutant putative miR-30 or two miR-203 binding sites in the CAR10 transcript (psiCHECK2-Mut30 and psiCHECK2-Mut203 respectively), mutant putative miR-30/203 binding sites of *SNAI1* 3′UTR or *SNAI2* 3′UTR (psiCHECK2-SNAI1 3′UTR Mut and psiCHECK2-SNAI2 3′UTR Mut respectively). (**H**) Luciferase activity in A549 cells cotransfected with miR-30/203 mimics and luciferase reporters containing *SNAI1*/*2* 3′UTR or mutant transcript. Data are shown as mean ± SEM; n = 3 independent experiments, two-tailed Student’s *t*-test. **p* < 0.05, ***p* < 0.01, and ****p* < 0.001, NS: no statistical significance.

**Supplementary Figure 6.** CAR10 promoted EMT through miR-30/203-*SNAI1*/*2* axis

(**A**) Overexpression of CAR10 enhanced the migration ability of A549 and PC9 cells, and this change was reversed by co-overexpression of miR-30 or/and miR-203. Scale bar: 200 μm. (**B**) Data from the wound-healing assay were quantified. Data are shown as mean ± SEM; n = 3 independent experiments, two-tailed Student’s *t*-test. (**C**) The mRNA levels of CAR10, *SNAI1* and *SNAI2* increased via inhibiting miR-30 or/and miR-203 in CAR10-knockout A549 cells. Data are shown as mean ± SEM; n = 3 independent experiments, two-tailed Student’s *t*-test. (**D**) Western blotting analysis of Snail and Slug protein levels in CRISPR-CTR or CAR10-knockout 549 cells with deletion of miR-30 or/and miR-203. (**E**) MiR-30/203 inhibitors and control inhibitor were transfected into CAR10-knockout A549 cells, and the cell invasion potential of these cells was detected in a Transwell assay (top). The cell migration ability assessed by a wound-healing assay (middle). Quantitation of the Transwell assay and wound-healing assay results are on right (bottom). **p* < 0.05, ***p* < 0.01, and ****p* < 0.001, NS: no statistical significance.
