# Supplementary figures and images for: Long noncoding RNA CAR10 promotes lung adenocarcinoma metastasis via miR-203/30/SNAI axis
Source: Oncogene. 2019 Jan 7;38(16):3061–76. doi: 10.1038/s41388-018-0645-x (PMC6484688; doi:10.1038/s41388-018-0645-x)

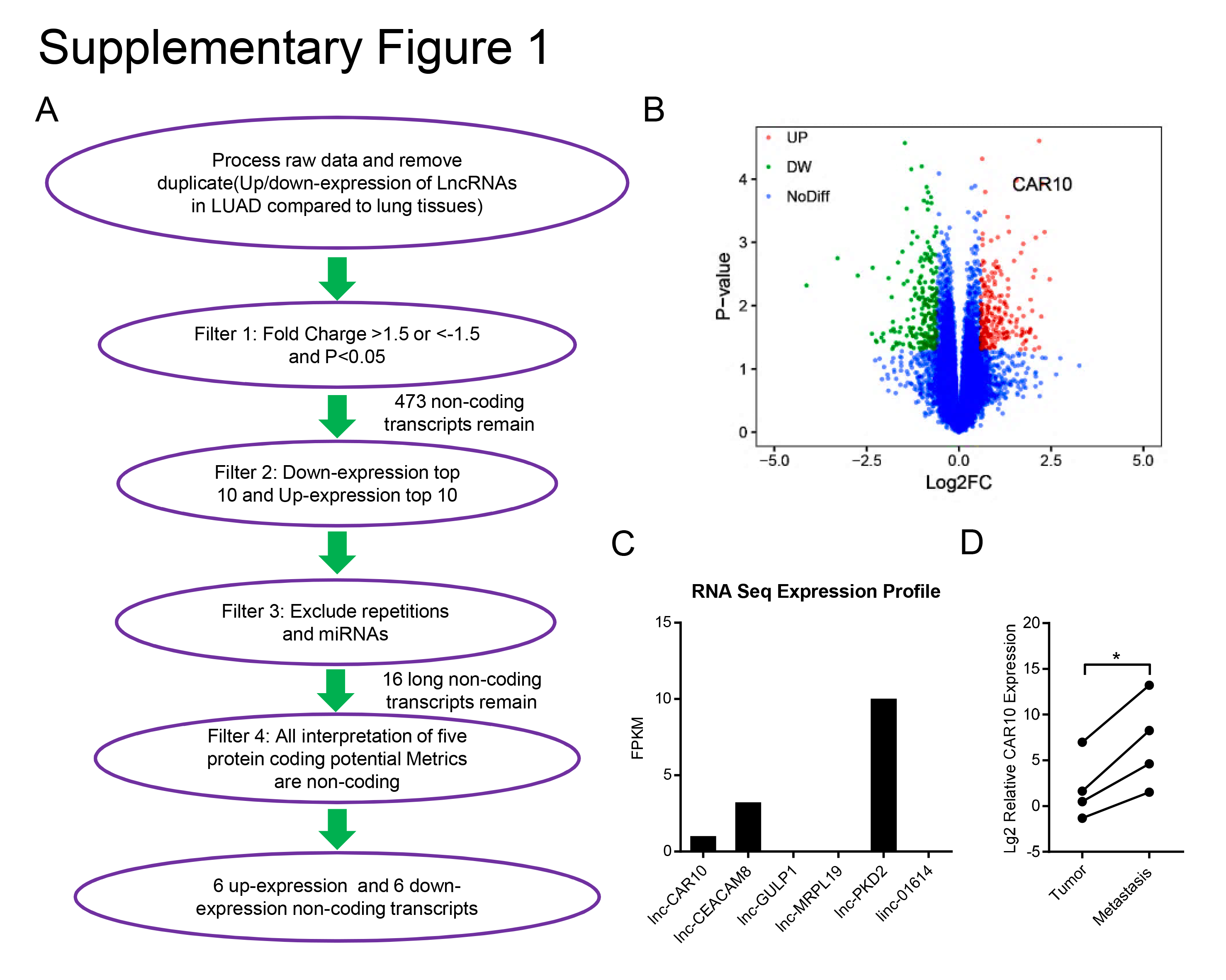

Supplement: Supplementary file 2 — Supplementary Figure 1 [file 41388_2018_645_MOESM2_ESM.tif]

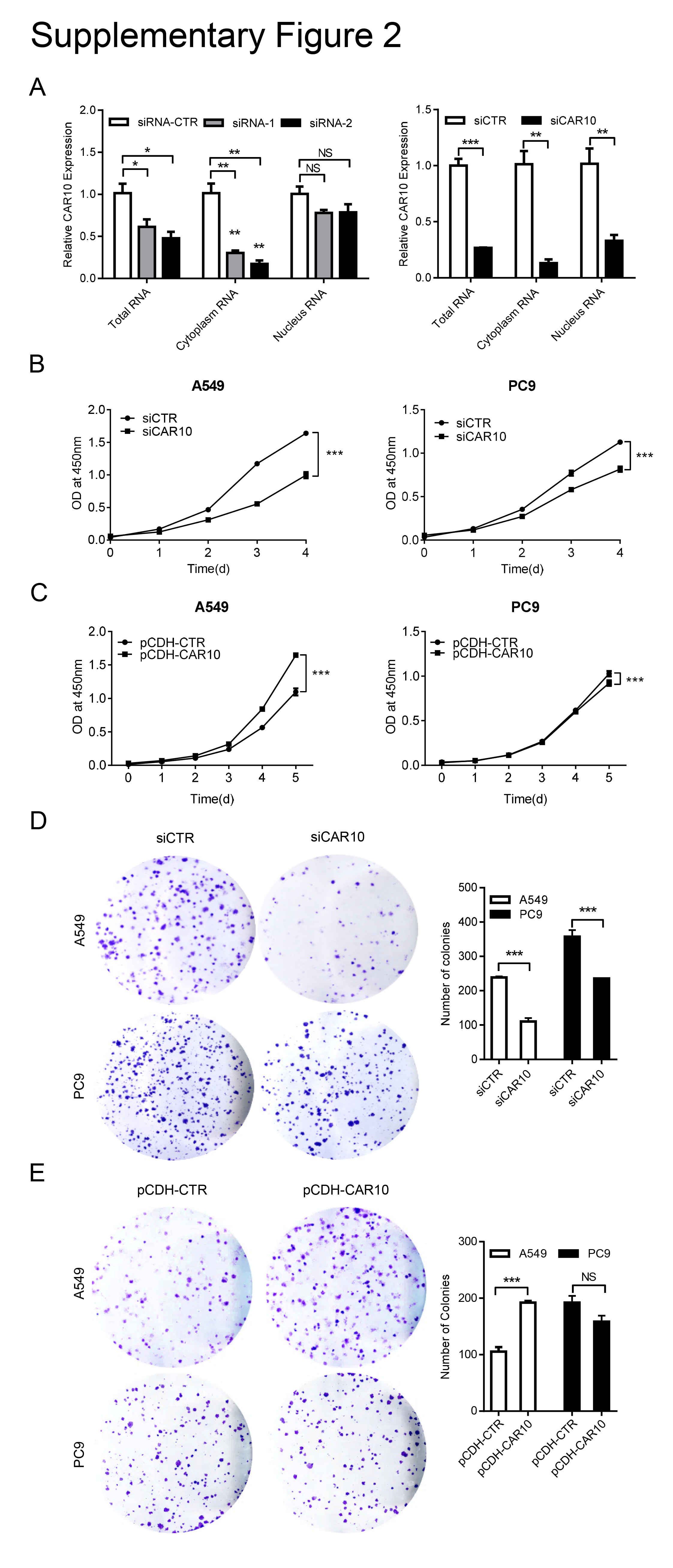

Supplement: Supplementary file 3 — Supplementary Figure 2 [file 41388_2018_645_MOESM3_ESM.tif]

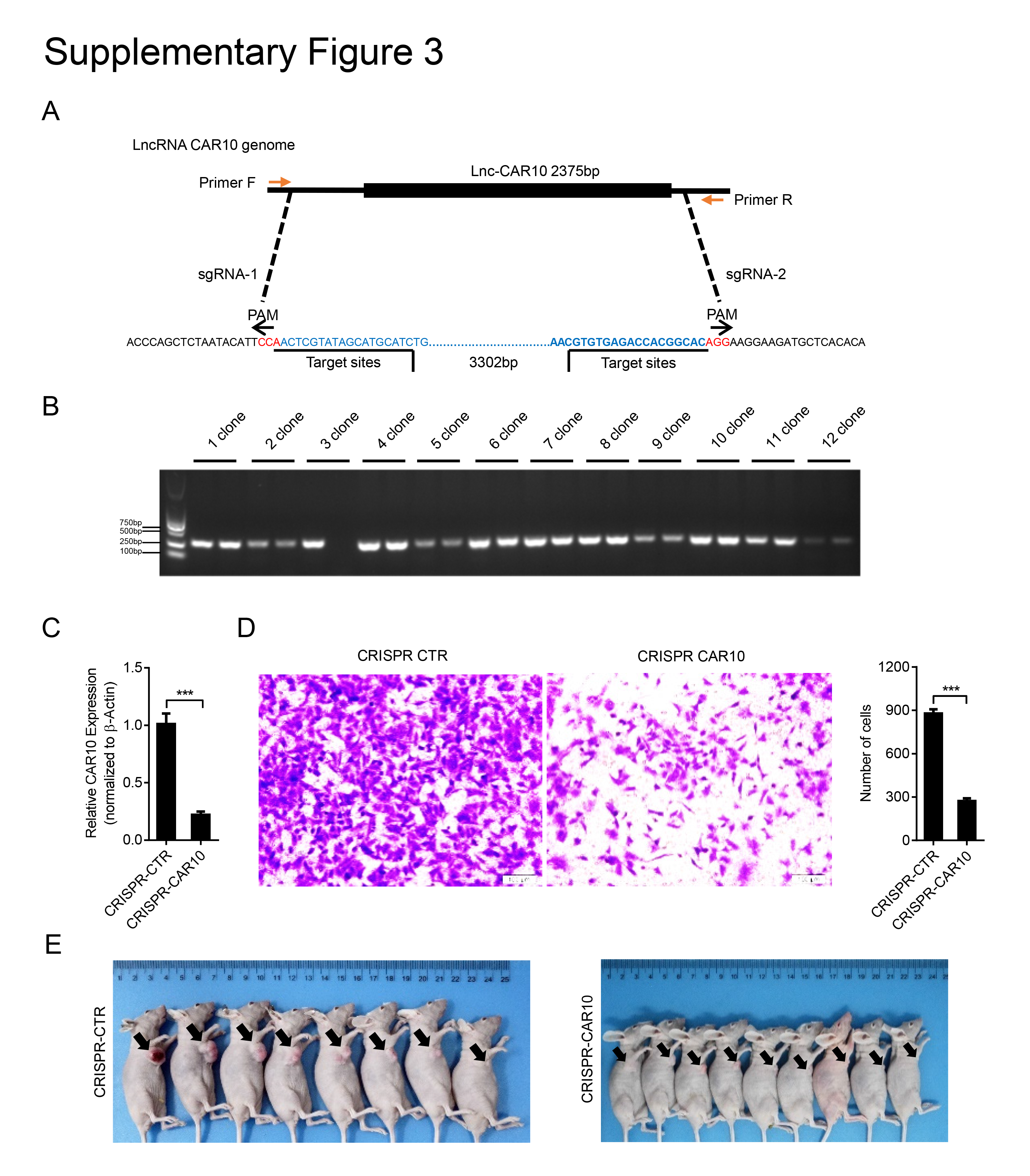

Supplement: Supplementary file 4 — Supplementary Figure 3 [file 41388_2018_645_MOESM4_ESM.tif]

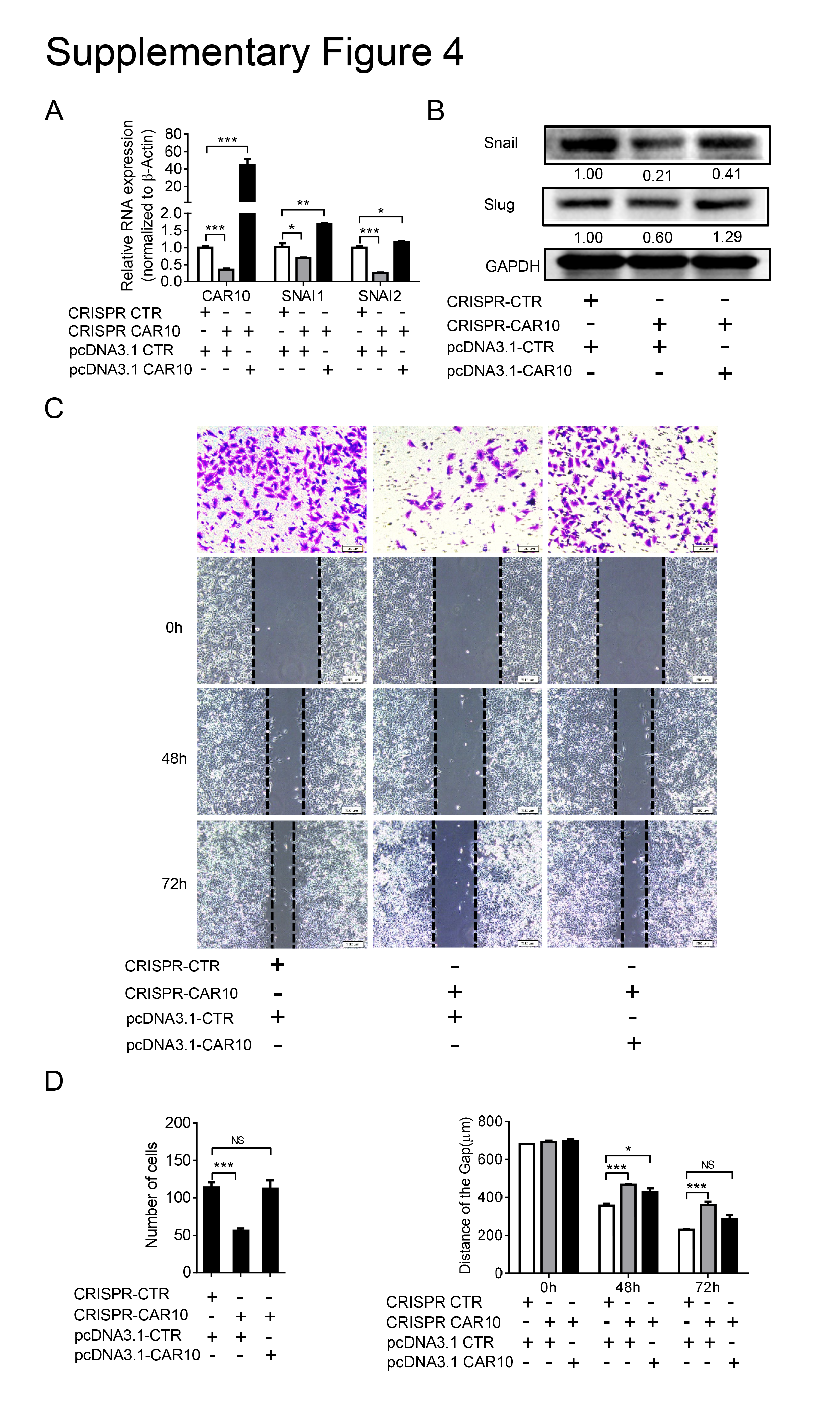

Supplement: Supplementary file 5 — Supplementary Figure 4 [file 41388_2018_645_MOESM5_ESM.tif]

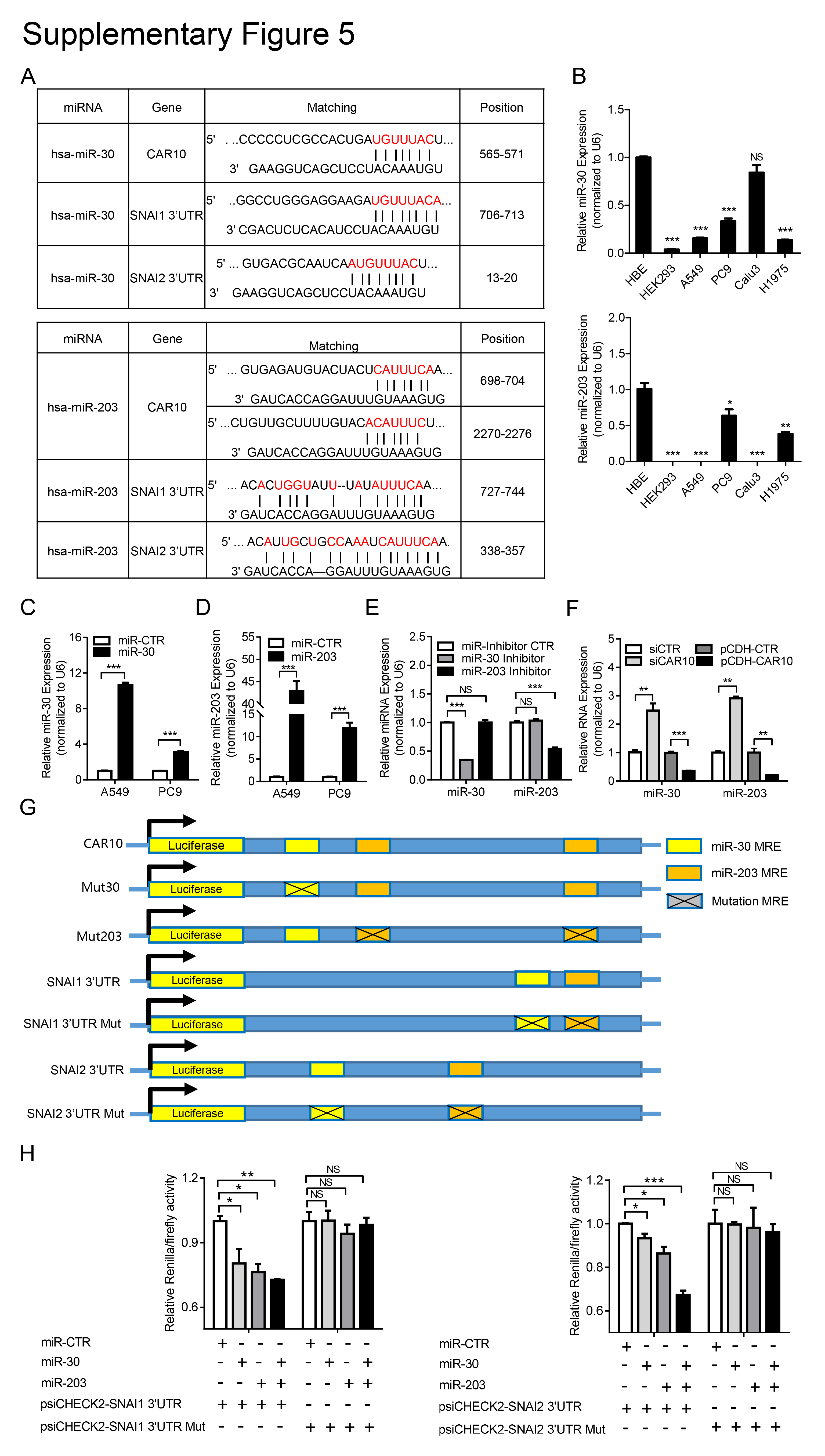

Supplement: Supplementary file 6 — Supplementary Figure 5 [file 41388_2018_645_MOESM6_ESM.tif]

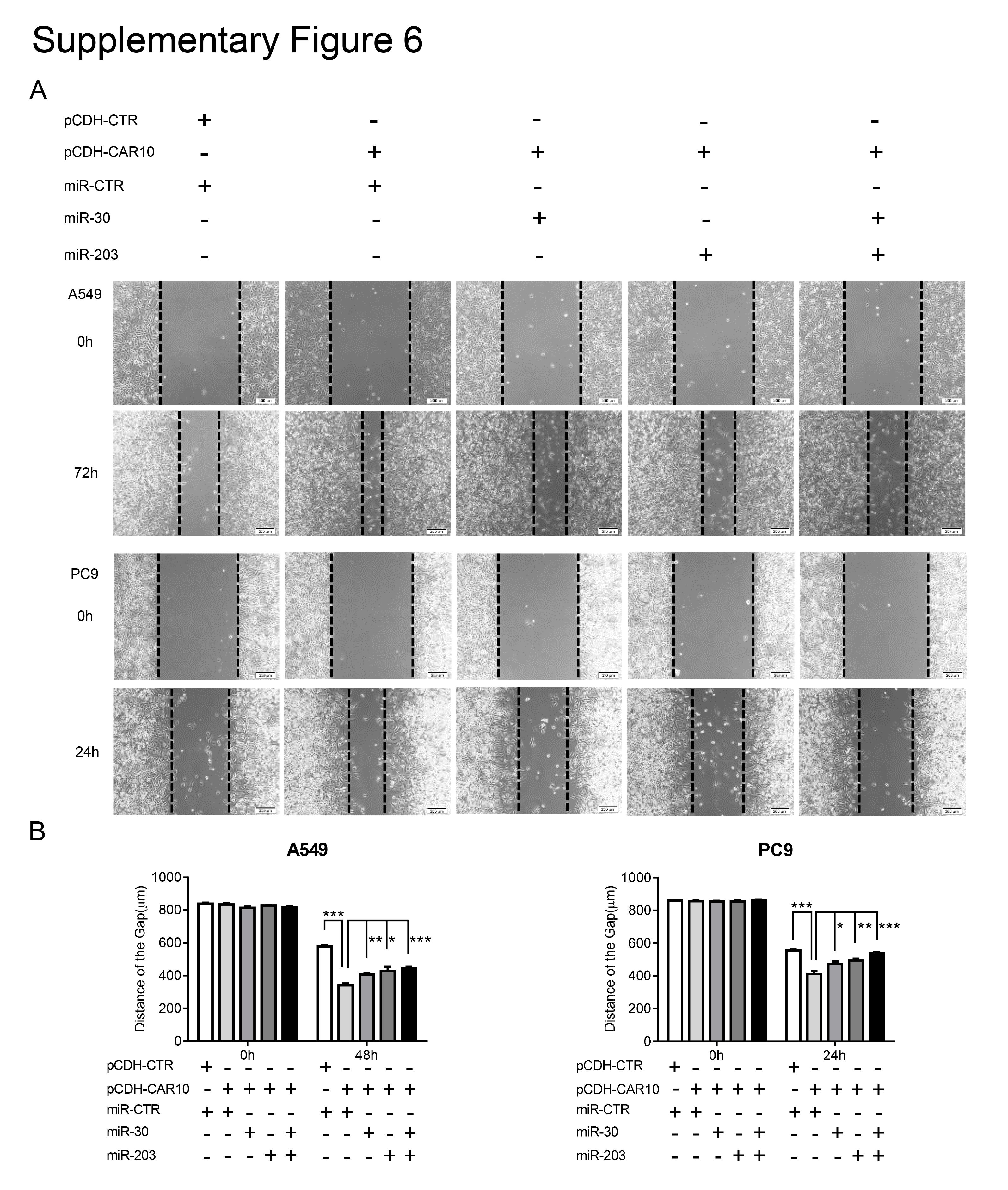

Supplement: Supplementary file 7 — Supplementary Figure 6A-B [file 41388_2018_645_MOESM7_ESM.tif]

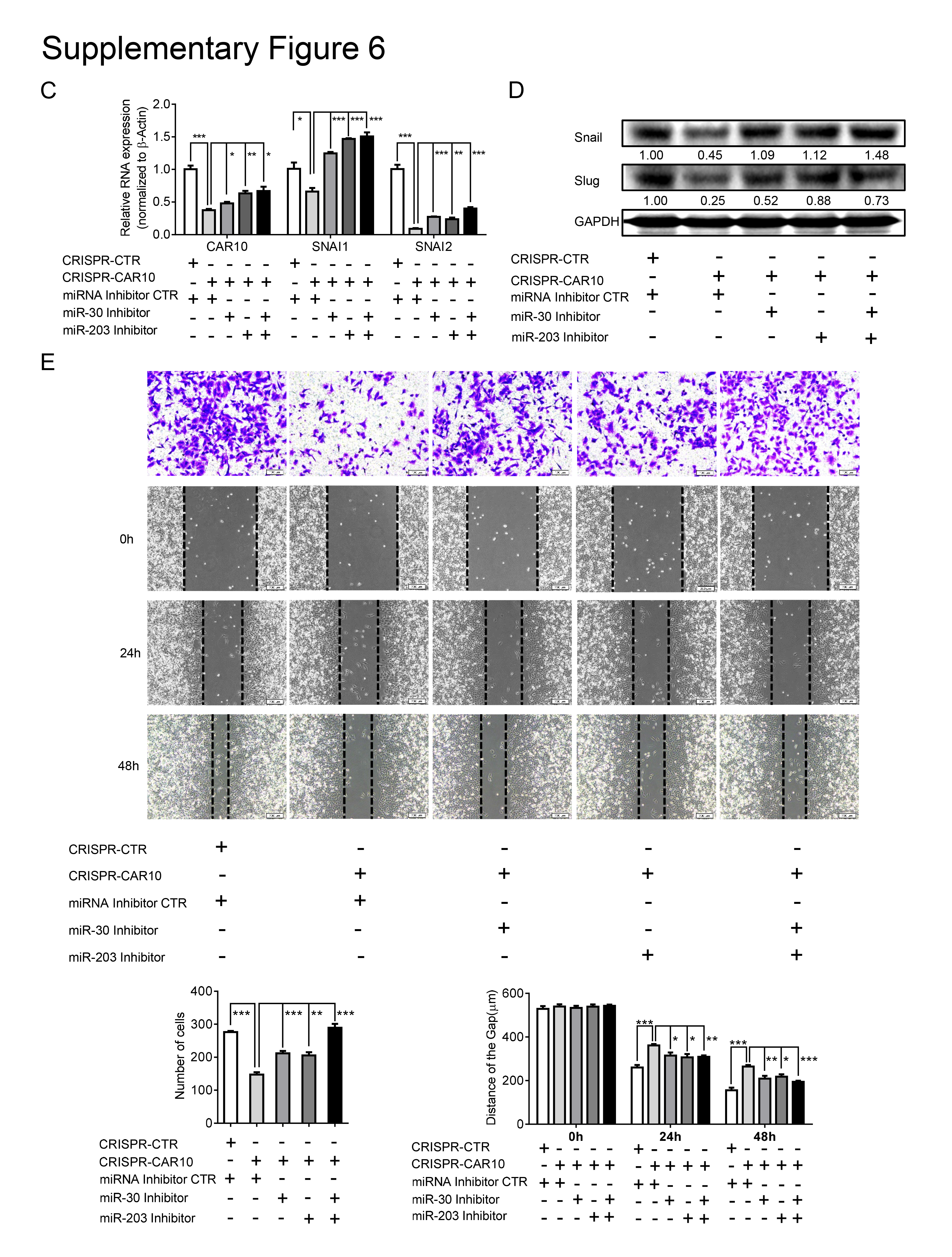

Supplement: Supplementary file 8 — Supplementary Figure 6C-E [file 41388_2018_645_MOESM8_ESM.tif]
